# Conservation in the Amazon rainforest and Google searches: A DCCA approach

**DOI:** 10.1371/journal.pone.0276675

**Published:** 2022-10-26

**Authors:** Eder J. A. L. Pereira, Paulo Ferreira, Ivan C. da Cunha Lima, Thiago B. Murari, Marcelo A. Moret, Hernane B. de B. Pereira

**Affiliations:** 1 PPG MCTI, Centro Universitário SENAI CIMATEC, Salvador, Bahia, Brazil; 2 Instituto Federal do Maranhão - IFMA, Bacabal, Maranhão, Brazil; 3 VALORIZA - Research Center for Endogenous Resource Valorization, Portalegre, Portugal; 4 Instituto Politecnico de Portalegre, Portalegre, Portugal; 5 CEFAGE-UE, IIFA, Universidade de Évora, Évora, Portugal; 6 Universidade do Estado da Bahia - UNEB, Salvador, Bahia, Brazil; 7 National Institute for Science and Technology-Petroleum Geophysics, INCT-GP, Salvador, Bahia, Brazil; 8 Pursuelife Consultancy on Applied Science, Salvador, Bahia, Brazil; 9 PPG GETEC, Centro Universitário SENAI CIMATEC, Salvador, Bahia, Brazil; Indiana State University, UNITED STATES

## Abstract

In this paper we analyze the descriptive statistics of the Google search volume for the terms related to the National Reserve of Copper and Associates (RENCA), a Brazilian mineral reserve in the Amazon of 4.6 million hectares, before and after the government signed the decree releasing it for exploration. First, we analyze the volume of searches for expressions related to RENCA in Google Trends using descriptive statistics; second, we assess the cross-correlation coefficient *ρ*_*DCCA*_, which measures the cross-correlation between two nonstationary time series across different time scales. After the government announced the release of the RENCA reserve, there was an increase in the average volume of Google searches for related terms, showing people’s concern about the announcement. By using the cross-correlation coefficient *ρ*_*DCCA*_, we identify strong cross-correlations between the different expressions related to RENCA in Google Trends. Our work shows the utility of Google Trends as an indicator of the perception of environmental policies. Additionally, we show that *ρ*_*DCCA*_ can be used as a tool to measure the cross-correlation between synonyms extracted from Google Trends for various time scales.

## Introduction

The Amazon rainforest contains more than half of the world’s rainforests and a quarter of all species on the planet and has a major role in determining global climate [1]. The National Reserve of Copper and Associates (RENCA) is an important Brazilian mineral reserve covering an area of over 4.6 million hectares inside the Amazon rainforest on the border between South and Southwest Amapá and Northwest Pará. This reserve was created during the military regime in 1984, and it was established by decree that the Mineral Resources Research Company (CPRM) would have the exclusive right to conduct geological research in the area.

On 08/23/2017, the Brazilian President Michel Temer signed the decree that allowed exploration of the RENCA. However, after a wide popular debate involving environmentalists, politicians, artists and the population, and after much criticism from abroad, the government canceled the decree [2, 3]. Initially, the decree signed on 08/23/2017 meant that RENCA would be opened up for exploration, setting off a new “gold rush”, which would jeopardize the survival not only of the local biome but also of the whole surrounding region. This event could lead to a phenomenon similar to that of iron ore exploration in the Carajás region [4], and in Pará, which caused irreversible environmental damage in the region.

However, even after the decree cancellation, approximately 7,900 square kilometers of Amazon forest were destroyed between July 2017 and July 2018, representing an increase of 13.7% over the same period of the previous year [5], and 30,901 fires were recorded in August 2019, the highest number of fires recorded in a month since 2006 [6]. The current Brazilian administration has always been vocal against environmental protections; it carried out a systematic dismantling of Brazilian environmental laws [7] and slashed Brazil’s environment budget [8]. The government also encourages agribusiness in the Amazon [9] and illegal mining on indigenous lands [10], and it is not committed to the goals of the Action Plan for the Prevention and Control of Deforestation in the Legal Amazon [11]. This situation has created concern among researchers about the current status of the forest [11–21].

Two indigenous lands (Paru dÉste and Waiõpi River), three protected areas (Jari Ecological Station, Tumucumaque Mountains National Park and Maicuru Biological Reserve) and four conservation units for sustainable use (Rio Cajari Extractive Reserve, Iratapuru River Sustainable Development Reserve, Amapá State Forest and Paru State Forest) were under threat. Their extinction could harm all of these surrounding Environmental Protection Areas (APAs). APAs are important [22, 23], since they cover an area of 13% of the planet (WDPA 2012). In addition, they are important for the maintenance, conservation and preservation of biodiversity, but they are under constant pressure because of competition for agricultural use [24]. Therefore, APAs show vulnerability due to frequent pressures, both social and political [25]. In addition, APAs are under threat through a process known as protected area downgrading, downsizing and degazettement (PADDD) [26].

Brazil has the largest APA system in the world, with 12.4% of the total or approximately 220 million hectares [27]. From that amount, indigenous lands, quilombola territories (areas reserved for the descendants of slaves), military areas and other types of parks and reserves known in Brazil as conservation units, which may be administered by the government, should be excluded from the PADDD process. [28] further highlight the importance of legal reserves for Brazilian biodiversity in relation to global climate change. They conclude that it is not necessary to deforest these reserves to increase agricultural production, since the country already has sufficient land available.

Google Trends (GT) is a tool developed by Google to measure the search for a certain term over the course of a month, week, day, hour and even minute. It reports the most searched terms on the site in a given period ranging from 0 to 100, where 0 means a very small or insignificant search, and 100 is the peak of searches for the term. In this way, Google has searched for 30 trillion URLs, which translates to over 100 billion queries per month worldwide [29, 30]. Any abnormal pattern found in GT may reveal a present behavior in society or even be used to anticipate future economic behavior [31]. Therefore, GT has been used to predict or understand web behaviors in various areas. In economics, GT data have already been used to predict financial markets [32], predict the value of bitcoin [33], diversify risk [34] and assess the relationship between searches for the term *Donald Trump* and fluctuations in the financial market [35]. There are also GT applications in cases of suicide and depression [36] and in the prediction of dengue cases [37].

A current discussion is whether interest in environmental conservation is shrinking or rising over time according to data provided by GT. Some studies find that interest in conservation issues is declining [38–40]. Following that line of thought, GT series were analyzed between 2004 and 2013 using the seasonal method Mann-Kendall test, which showed a decrease in public interest in the terms *climate change, ecosystem services, deforestation, orangutan, invasive species* and *habitat loss* [41]. [42] showed that interest in the environment is not decreasing but increasing. To do so, the author compared six terms related to the environment and found that the search for terms related to *internet, science* and *leisure* is increasing.

In this article, we use descriptive statistics to measure the volume of searches for terms related to RENCA, after the decree by which former president Michel Temer announced the release of this reserve for exploration. Another objective is to propose a metric for the use of synonymous expressions of GT through the use of the cross-correlation coefficient (*ρ*_*DCCA*_) proposed by [43].

## Materials and methods

We extracted the data from GT. We took a weekly scale and considered only Brazil since the aim was to verify the Brazilian population’s interest in the debate regarding the release of RENCA. To choose the terms in GT, we considered the importance of cultural and linguistic aspects, including the influence of different languages [44]. That said, for this work, we chose the following expressions in Brazilian Portuguese to refer to RENCA: *Renca Amazônia, Renca Reserva and Renca Decreto*. The term *Renca* had to be avoided because in Chile, there is a province with this name. In addition, we used descriptive statistics to observe the data using average, median, minimum, maximum and standard deviation to capture a possible increase in the population’s interest in the reserve after the approval for exploration was announced.

**Fig 1:**
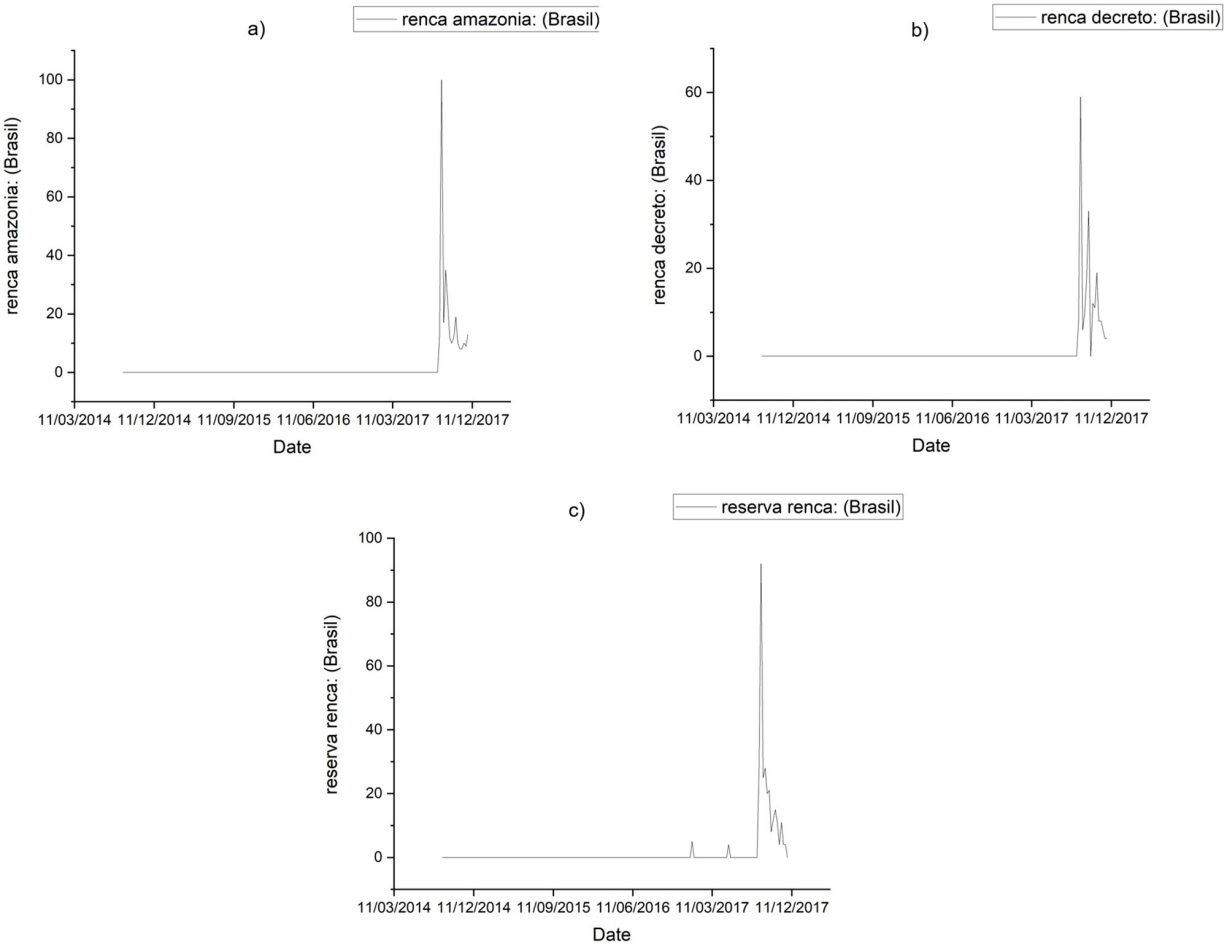
GT searches for RENCA terms. Panel a) shows searches on GT for *Renca Amazônia*, Panel b) shows *Renca Decreto* and Panel c) shows searches for *Reserva Renca* between Aug. 24^th^, 2014 and Nov. 26^th^, 2017.

The period between 08/24/2014 and 11/26/2017 was analyzed, considering the date of the announcement of the release for exploration of RENCA on 08/23/2017 as a limit for the ***ex ante*** periods (prior to the release announcement 08/24/2014 to 08/23/2017) and ***ex post*** (after release announcement 08/24/2017 to 11/26/2017). Fig 1 shows that before the announcement of the decree, searches for terms related to RENCA were practically zero. However, after that, GT searches for expressions related to the reserve rose suddenly during the week of the announcement (08/20/2017 to 08/27/2017), and even when the government canceled the decree on 09/26/2017, searches remained high during the following months. All raw data are available from the Harvard Dataverse [45].

To help demonstrate that the expressions chosen (*Renca Amazônia, Renca Reserva and Renca Decreto*) correspond to the term RENCA, we calculated the cross-correlation coefficient to analyze all cross-correlations between the terms representing RENCA. If the cross-correlations between these expressions representing RENCA are high, this helps to demonstrate that they are related to the same term. Then, we calculated the cross-correlation of the terms corresponding to RENCA with three random terms in Brazilian Portuguese, extracted from GT for Brazil for the same dates. The random terms chosen were *star wars, oil (petróleo)* and *soccer (futebol)*. The objective of this comparison is to show that, independent of the time scale, the correlation between the representative terms of GT and the random expressions are not significant, since there is no relationship between searches of these terms in Google and RENCA. The use of correlation calculations to demonstrate which expressions refer to the same theme was proposed by [46], who calculated the existing correlation for relative search volume (RSV) on the internet corresponding to the protected wetland areas (PWAs) in South Korea using Spearman nonparametric correlation analysis. Next, we demonstrate the detrended cross-correlation coefficient [43].

$$\begin{array}{c} \rho_{DCCA}=\frac{F_{DCCA}^{2}}{F_{DCCA}\left(x_{i}\right)F_{DCCA}\left(y_{i}\right)}. \end{array}$$ (1)

The detrended cross-correlation coefficient is a method to quantify the level of correlation between two nonstationary time series [43]. This method is based on detrended fluctuation analysis (DFA) [47] and detrended cross-correlation analysis (DCCA) [48]. Below, we present the algorithm:

Step I: considering two time series, {*x*_*t*_} and {*y*_*t*_}, with *t* = 1, 2, …, *N*, where (*N* time series length). Then, we integrate the time series to obtain two new time series:
  $$\begin{array}{c} xx_{k}=\sum_{t=1}^{k}y_{t},k=1,2,...,N \end{array}$$ (2)
Step II: we divide these two integrated time series, {*xx*_*k*_} and {*yy*_*k*_}, into (*N*−*s*) overlapping boxes of equal length *s*, with 4≤s≤N4 .
Step III: we calculate the local trend of each of the boxes by a least-squares fit of each series, *xP*_*i*_(*k*) and *yP*_*i*_(*k*). Next, we calculate the covariance of the residuals in each box by:
  $$\begin{array}{c} f^{2}{}_{xy}(s,i)=\frac{1}{s+1}\sum_{k=i}^{i+s}(xx_{k}-xP_{i}(k))(yy_{k}-yP_{i}\left(k\right))\; \end{array}$$ (3)
Step IV: thus, we calculate the average over all overlapping boxes to obtain the new covariance function:
  $$\begin{array}{c} F^{2}{}_{xy}(s)=\frac{1}{\left(N-s\right)}\sum_{i=1}^{N-s}f^{2}{}_{xy}(s,i) \end{array}$$ (4)
Step V: finally, we calculate the cross-correlation coefficient *ρ*_*DCCA*_ using the ratio of the covariance function, i.e.,
  $$\begin{array}{c} \rho_{DCCA}\left(s\right)=\frac{F^{2}{}_{xy}(s)}{F_{xx}(s)F_{yy}\left(s\right)} \end{array}$$ (5)

This cross-correlation coefficient depends on the length of each box (the time scale). One of the advantages of this cross-correlation coefficient is that it measures the correlations between two time series at different time scales. The DCCA cross-correlation coefficient ranges from −1 ≤ *ρ*_*DCCA*_ ≤ 1, where 1 means perfectly correlated, -1 means completely anticorrelated and 0 means there is no cross-correlation.

$F_{DCCA}^{2}$ measures the long-range correlation between two different variables, as described by detrended cross-correlation analysis [48], while *F*_*DCCA*_(*x*_*i*_) and *F*_*DCCA*_(*y*_*i*_) are based on detrended fluctuation analysis [47]. They were combined by [43] as covariance and variance variables, as in the traditional Pearson correlation coefficient.

This is an efficient correlation coefficient (see, for example, Ref. [49]) used to estimate correlations between variables using different time scales; it could also be used in nonstationary data. We use the procedure of [50] to test the significance of the correlation. *ρ*_*DCCA*_ varies in the interval −1 ≤ *ρ*_*DCCA*_ ≤ 1, where 1 means perfect cross-correlation, -1 means perfect cross-correlation, and 0 means no correlation exists. In terms of intensities, the categories and values are as follows: weak (0.00 to 0.33 or 0.00 to -0.33), medium (0.33 to 0.66 or -0.33 to -0.66), and strong (0.66 to 0.99 or -0.66 to -0.99) [51, 52].

This approach has applications in finance, [53–59], climatology [60] and criminology [61]. An advantage of this approach compared to other correlation coefficients, such as Pearson correlation coefficient, is that it is a multiscale correlation method and it is possible to obtain correlations for several timescales, both linear and nonlinear [62]. Moreover, multiscale methods have been recommended to work with big data [63], as it is in the case of data extracted from GT.

In the environmental sciences, *ρ*_*DCCA*_ has been used to measure the population’s interest in environmental conservation. According to Soriano-Redondo et al. [64], the use of GT data in the environmental sciences can serve as a parameter to measure interest in environmental conservation projects or in ecological thinking. [65] reported that there is a strong relationship between the public interest in protected areas (as measured by GT) and its visibility on the internet, noting that more studies should be conducted to prove this relationship. Therefore, public perception of environmental conservation was monitored using internet-based methods, in particular offsite and onsite metrics, arriving at the conclusion that these methods can capture the perception of environmental conservation both in time and space [64].

## Results and discussion

Table 1 shows the descriptive statistics of the GT searches by the terms corresponding to RENCA: *Renca Amazônia*, *Renca Decreto* and *Renca Reserva*. Before the release of RENCA for exploitation was announced, the average of the searches by the three terms in GT was almost insignificant, with the median being equal to zero.

**Table 1:** Descriptive statistic for searches on GT for Renca Amazônia in Brazil, before (*ex ante*) and after (*ex post*) environmental policy implementation.

Situations	Searches on Google Trends				
Ex ante	Average	Median	Minimum	Maximum	Standard
*Reserva Renca*:	0.236	0	0	28	2.287
*Renca Decreto*:	0.051	0	0	8	0.638
*Renca Amazônia*:	0.0764	0	0	12	0.958
Ex post	Average	Median	Minimum	Maximum	Standard
*Reserva Renca*:	18.214	11.5	0	92	22.858
*Renca Decreto*:	14.143	9	0	59	15.321
*Renca Amazônia*:	20.5	12	8	100	24.073

However, after the decree, there was an increase in searches for all terms related to the reserve. Considering the term *Renca Amazônia*, the average of the searches in GT went from 0.07 to 20.5, that is, an increase of 29,185%. The average GT searches for the term *Renca Decreto* rose from 0.05 to 14.1, representing an increase of 28,900%. The average search for the term *Renca Reserva* in Google went from 0.23 to 18.21, meaning an increase of 7,817%. After the release, all terms related to RENCA had a significant increase in Google searches in both the average and the median. Regarding the maximum reached by the searches in GT, highlighted are the terms *Renca Amazônia*, where searches reached a maximum of 100, and for the term *Renca Reserva*, a maximum of 92, showing a considerable peak in the searches for these terms. The results show a substantial increase in the population’s interest in the reserve after the decree, which lasted for the next three months, even after the government stepped back and canceled the decree releasing the reserve. This increase in interest by web-users in Brazil, together with criticism both nationally and abroad, influenced the Brazilian government to revert its decision to release the RENCA for exploration on September 26, 2017.

**Fig 2:**
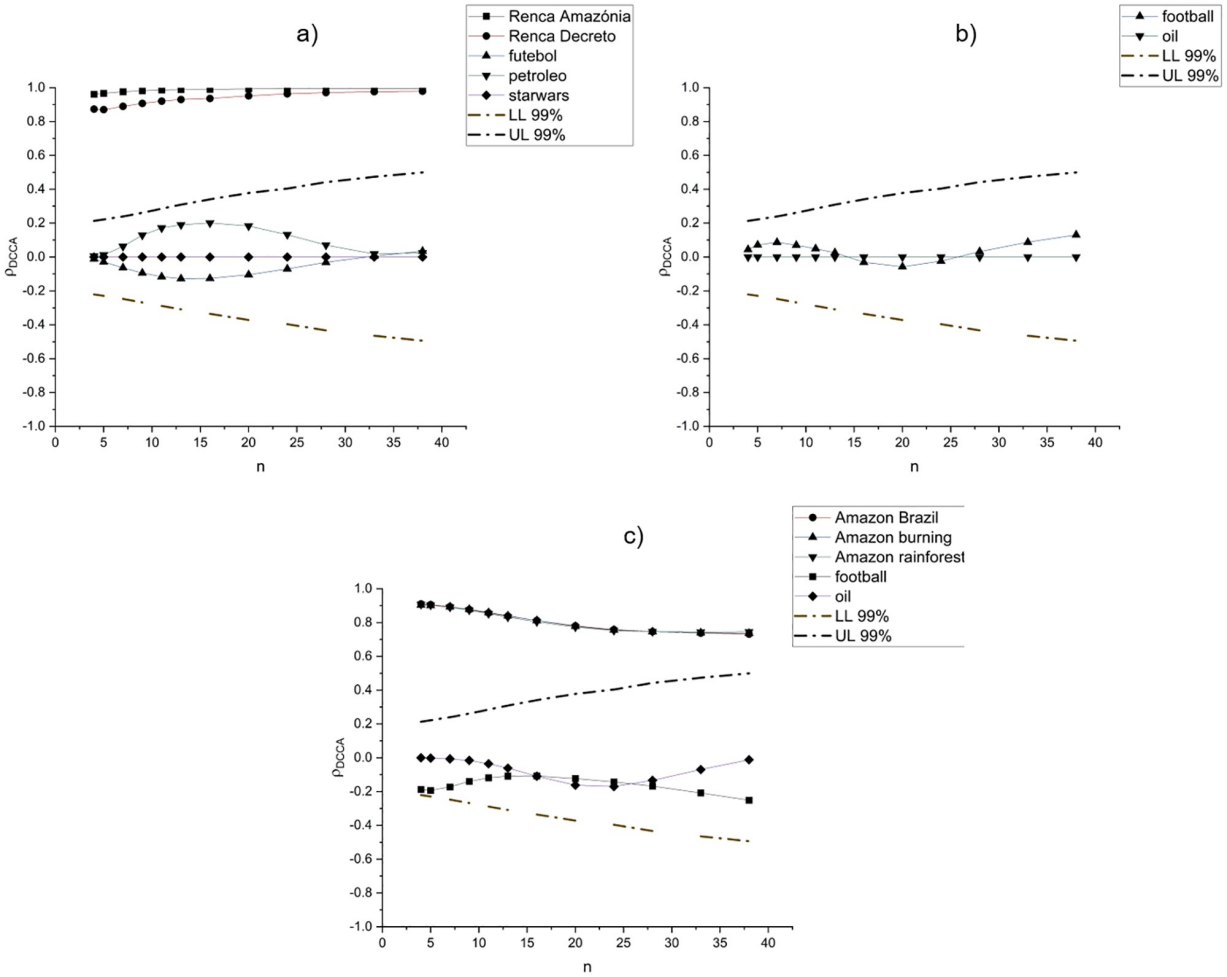
DCCA coefficient for all RENCA terms. Panel a) shows the correlations between *Renca Reserva* and *Renca Amazônia, Renca Decreto, futebol* (football, in Portuguese), *petroleo* (oil, in Portuguese) and *starwars*; Panel b) shows the correlations between *Renca Reserva* and the terms *football and oil*; Panel c) shows the correlation coefficient between the term *Amazon fire* and the terms *Amazon Brazil, Amazon burning, Amazon rainforest, football* and *oil*. The terms *Amazon Brazil, Amazon burning, Amazon rainforest* presented similar results and are visually overlaid in Panel c. In all the panels, dashed lines show the critical values with a significance level of 99%, used to analyze the statistical significance of the correlation coefficients.

Looking at the cross-correlations between the terms related to RENCA and the random terms, Fig 2a shows that the correlation of the terms extracted from GT, *Renca Reserve* and the other terms related to the reservation (*Renca Amazônia* and *Renca Decreto*) peaked above 0.8 (for all the time scales), and it can be observed that the larger the time scale, the closer the value comes to 1. The correlation between the volume of searches for the terms *Renca Reserva* and *Renca Amazônia* on Google was higher than that for the terms *Renca Reserva* and *Renca Decreto*. To corroborate the results, we also measured the cross-correlation coefficient between the terms related to the reserve and the random terms (*soccer, oil* and *star wars*) and found that the statistical relationship between these random indicators is not statistically significant.

A strong intensity value was found for the cross-correlations between the volume of searches on Google for the terms representing RENCA and an insignificant value between them and the volume of searches on Google for the random terms. In this way, calculating the cross-correlation can make the choice of terms extracted from GT more robust. This is not the only option, as other cultural and linguistic factors could be considered. Nevertheless, any method that helps reduce uncertainty in the choice of correlated terms is welcome to make the analysis more robust.

For robustness, we performed three other different analyses: first, a similar analysis using similar terms, but in English, namely, the correlation between *Renca Reserva, football* and *oil*; second, an analysis now using terms associated with another environment episode, namely, the recent fires in the Amazon forest, using the terms *Amazon fire, Amazon rainforest, Amazon burning, Amazon Brazil, football, oil* and *starwars*); and third, we used the Pearson correlation coefficient for the different set of words used in the previous analyses. With the first two approaches, we want to show that the DCCA correlation coefficient can detect different types of correlations, and with the last, we check the difference in results between both correlation coefficients, showing the advantages of the DCCA.

We confirmed that there is a near zero correlation between the random terms (with *oil*, the correlation is, in fact, null), as shown in Fig 2b. The second approach was to make a similar analysis, with a new event, with the results presented in Fig 2c. Considering the term *Amazon fire* as the basis, we confirm a positive and significant correlation between the terms related to *Amazon*, noting that the correlation levels are very similar. Once again, the correlation with other random terms was nonsignificant.

Additionally, for robustness, we calculated the Pearson correlation coefficient for the first set of terms (the set including Portuguese terms) and for the last set of terms (including English terms). Calculation of the Pearson coefficients is possible because the series are stationary, according to the Augmented Dickey-Fuller unit root tests (results available upon request). The results of the correlations are presented in Table 2, and for comparison purposes, we calculated the average of the DCCA correlation coefficient.

**Table 2:** Comparison between Pearson and DCCA correlation coefficients.

Basic term: *Reserva Renca*	*Renca Amazonia*	*Renca Decreto*	*futebol*	*petroleo*	*starwars*
*ρ*_*DCCA*_ (mean)	0.986 ± 0.0119	0.931 ± 0.0391	-0.061 ± 0.0547	0.100 ± 0.0757	0.000 ± 0.0000
*ρ* Pearson**	0.967	0.900	-0.022	0.081	-0.027
Basic term: *Amazon fire*	*Amazon rainforest*	*Amazon burning*	*Amazon Brazil*	*Football*	*Oil*
*ρ*_*DCCA*_ (mean)	0.821 ± 0.0683	0.820 ± 0.0669	0.819 ± 0.0658	-0.160 ± 0.0446	-0.065 ± 0.0640
*ρ* Pearson**	0.843	0.841	0.844	-0.180	-0.013

**significance level of 0.01.

The coefficients show similar values, confirming the robustness of the DCCA correlation coefficient. As previously mentioned, this coefficient can detect correlations for different time scales, while the Pearson coefficient is only contemporary. For example, looking at Fig 2c, we can see a slight decrease in the coefficient of the terms related to the Amazon, showing that in the long run, the correlations are lower than in the short run. This could be an important indicator, for example, of the possibility of some subjects falling by the wayside as long as time passes.

## Conclusions

Google Trends can be an important means of highlighting governmental decisions that may be contrary to the public interest, and environmental preservation is one such case. Thus, scientists, environmentalists and policy-makers should make use indicators as warning signs expressing the status and evolution of the public interest and concern about conserving biodiversity [66]. When the RENCA mineral reserve was declared open to exploitation, there was an average increase of 29,185% in the searches for the expression *Renca Amazônia*, extracted from Google; for the expression *Renca Decreto* this increase was 28,900% and for the expression *Reserva Renca* it was 7,817%. These results demonstrate increased interest among the Brazilian population in conserving the reserve, putting the government under pressure to revert the decision to open RENCA for exploration.

Another relevant result is that the expressions associated with RENCA on GT (*Renca Amazônia, Renca Decreto* and *Renca Reserve*) presented a value for the cross correlation coefficient above 0.8 (for all time scales), and the largest value was nearly 1. This result demonstrates that they are related to the same theme. In this context, the use of the method presented here can help choose expressions in GT that deal with the same theme because if a term has a strong cross-correlation with other terms, it can indicate that these terms are synonyms.

The attempts of the current Brazilian administration to relax forest regulation, in addition to diminishing tree cover in the Amazon, lead to increased attention on how to introduce and maintain policy reforms for the preservation of Brazilian forests [67]. Interest has increased in topics such as opening indigenous lands to exploitation, reducing investment in forest watchdog agencies, exploring new areas without environmental licensing [7, 68] and opening several areas located within important environmental protection areas in the Amazon to mining [8, 10].

Therefore, increased interest among people in Brazil in conserving RENCA was reflected in the increase in searches for terms related to the reservation in Google. It is reiterated that, for the Amazon, it is of the utmost importance that the population expresses its opinion about themes related to environmental conservation that involve the forest and its biome. This is a direct way of forcing the country’s authorities to think of laws or projects that contribute to its preservation. GT may be used as an indicator that can monitor part of the population’s opinion on the conservation of world biodiversity.
